# Tobacco and alcohol use in rural elderly Indian population

**DOI:** 10.4103/0019-5545.43050

**Published:** 2005

**Authors:** Anil Goswami, V.P. Reddaiah, S.K. Kapoor, Bir Singh, S.N. Dwivedi, Guresh Kumar

**Affiliations:** * Supervising Medical Social Service Officer, Centre for Community Medicine, All India Institute of Medical Sciences; ** Ex-Professor and Head, Centre for Community Medicine, All India Institute of Medical Sciences; *** Professor, Centre for Community Medicine, All India Institute of Medical Sciences; **** Professor, Centre for Community Medicine, All India Institute of Medical Sciences; ***** Additional Professor, Department of Biostatistics, All India Institute of Medical Sciences; ****** Scientist, Department of Biostatistics, All India Institute of Medical Sciences

**Keywords:** Tobacco use, alcohol use, elderly, rural

## Abstract

**Background:**

Tobacco and alcohol use are serious health problems. Studies focusing on problems associated with tobacco and alcohol use in the elderly are limited.

**Aim:**

To find out the prevalence of tobacco and alcohol use among rural elderly population.

**Methods:**

This cross-sectional study was conducted in the intensive field practice area of the Comprehensive Rural Health Services Project in Ballabgarh in Faridabad, Haryana, a rural field practice area of the Centre for Community Medicine, All India Institute of Medical Sciences, New Delhi. The sample was selected using stratified random cluster sampling. The participants were ≥60 years of age at the time of interview. Data on tobacco and alcohol use pattern of 1117 elderly were collected during the interview.

**Results:**

The prevalence of smoking was 71.8% in men (*n*=490) and 41.4% in women (*n*=497). Among men smokers, 41.5% were light smokers (≤5 *beedis*/day), 42.9% were moderate smokers (6–20 *beedis*/day) and 15.6% were heavy smokers (>20 *beedis*/day). Among women smokers, 71.8% were light smokers, 23.8% were moderate smokers and 4.4% were heavy smokers. Regular alcohol intake was seen in 16.3% of the men compared with 0.8% of the women.

**Conclusion:**

The finding of a high prevalence of smoking and alcohol consumption among men in this rural population of India is of serious concern and therefore needs remedial measures.

## INTRODUCTION

Tobacco and alcohol use are serious public health problems in many countries including India because of the associated health hazards. Smoking causes a vast spectrum of diseases, many of which could result in death. There are over 50 diseases that are caused, increased or exacerbated by smoking.^1^ Globally, approximately, 47% of men and 12% of women smoke. Available data suggest that in developing countries, 48% of men smoke compared with 7% of women, while in developed countries, 42% of men and 24% of women smoke.^2^

The World Health Organization (WHO) estimates that 4.9 million deaths (8.8%) and 59.1 million disability-adjusted life-years (DALYs) (4.1%) are attributable to tobacco every year. Unless the current trends are reversed, the figure is expected to raise to 10 million deaths per year by the 2020s or early 2030s, with 7 million of these deaths occurring in developing countries, mainly in China and India.^2^ Worldwide, about one-fifth of all deaths attributed to tobacco occur in India, i.e. more than 8,00,000 people die and 12 million become ill as a result of tobacco use every year. The deaths attributed to tobacco in India are expected to rise from 1.4% of all deaths in 1990 to 13.3% in 2002.^3^

According to the *World Health Report 2002*,^4^ among industrialized countries, where smoking is common, the habit is estimated to cause over 90% of lung cancer in men and about 70% of lung cancer among women. In addition, in these countries, the attributable fractions are 56%–80% for chronic respiratory disease and 22% for cardiovascular diseases.

Though many of the alcohol-related health effects have been recognized recently, the use of alcohol in human life is very old. There is a casual relationship between alcohol consumption and more than 60 types of diseases and injury. Worldwide, alcohol is estimated to cause about 20%–30% of oesophageal cancer, liver cancer, cirrhosis of the liver, homicide, epilepsy and motor vehicle accidents. Worldwide, 1.8 million deaths and 58.3 million DALYs are attributed to the use of alcohol. Thus, consumption of alcohol is a cause of concern to minimize these problems.^2^

The different forms of tobacco used in India are smoking tobacco such as *beedi,* cigarette, *hookah* (hubble-bubble) and cigar; chewable tobacco such as *gutka, khaini;* applying tobacco such as *gul*; and other forms such as tobacco-containing tooth powder/tooth paste and snuff.

Most of the studies on tobacco and alcohol use focus on the prevention of problems related to the use of these substances in adolescents and youngsters. However, studies focusing on the elderly are comparatively infrequent. Thus, there is a need to study this aspect in India. Some community-based rural studies^5^,^6^ have shown the prevalence of tobacco and alcohol use as 31%–42% and 10%, respectively in those ≥60 years of age. As almost three-fourths of the elderly people live in rural India, there is a need to know the prevalence of tobacco and alcohol use in these areas to plan for educational efforts directed towards this group. Cessation of tobacco and alcohol use can still slow down the incidence of cardio-vascular, pulmonary or malignant diseases, and prevent substance-induced problems. This study estimates the extent and pattern of tobacco and alcohol use among rural Indian elderly population.

## METHODS

This study was conducted in the Intensive Field Practice Area (IFPA) of the Comprehensive Rural Health Services Project (CRHSP) in Ballabgarh, Faridabad, Haryana. This is a rural field practice area of the Centre for Community Medicine (CCM), All India Institute of Medical Sciences (AIIMS), New Delhi. The data were collected from January 1998 to December 1999. Twenty-eight villages with a population of 69,995 were covered during this period. Health services are provided by two primary health centres (PHC) in Dayalpur and Chhainsa, covering 8 subcentres (SCs)—6 non-PHC and 2 PHC SCs. The demographic data of the entire population are available in electronically stored database, which are updated annually. The sample was selected using stratified random cluster sampling. To take a representative sample, the SCs were stratified on the basis of the availability of a health facility, i.e. PHC SCs (2) and non-PHC SCs (6). The sample SCs were selected randomly by draw of lots, i.e. one SC out of the 2 PHC SCs and one SC out of the 6 non-PHC SCs.

All the villages in the selected two SCs were included in this cross-sectional study. Each village served as a cluster and all the elderly people in the village (≥60 years of age) were studied. The participants had been residents of the area for at least six months. A computerized list of the elderly was obtained from a database of the study area. If participants were found to be absent on one visit, another visit was made within 7 days. If they could not be contacted even after two visits, they were excluded from the study. Personal interviews were conducted in the local language in the homes of the respondents. An informed verbal consent was taken from each participant. When necessary, subjects were referred for further examination/investigation and treatment. The approval of the ethics committee was taken for conducting this study.

Smoking was defined according to the WHO classification. Ever-smokers were defined as persons who had ever smoked for at least 6 months. Current smokers were persons smoking tobacco at the time of the survey. Among current smokers, daily or regular smokers were persons smoking at least 1 *beedi*/cigarette every day. Any respondent smoking 5 *beedis*/cigarettes per day was taken as a light smoker, between 6 and 20 as a moderate smoker, and >20 as a heavy smoker.

Alcohol users were classified as past and current users. Past users were defined as persons who had consumed alcohol at least once during their lifetime but had not done so for a period of one year preceding the survey. Current users were those who had consumed alcohol at least once during the past one year preceding the time of the interview. Current users were further classified as occasional and regular users. Occasional users were defined as persons who consumed alcohol less often than once a week. Individuals who had consumed alcohol at least once a week, or several times a week, or on a daily basis, and more than one time in a day were classified as regular users.

The data were collected using a semi-structured interview schedule adapted from standardized schedules.^8^,^9^ Detailed information was collected regarding basic demographic characteristics, living conditions, and tobacco and alcohol use. The first author [AG] took all the interviews and measurements.

The data were analysed using the Epi Info 6.04 d and the SPSS version 7.5 software. The chi-square test was used to compare proportions.

Of the total population of 17,795 in 7 selected villages, 1117 were elderly persons (6.3% of the total population). Out of these, only 987 (88.4%) could be interviewed. Twelve of them refused to cooperate (1.1%) and the remaining 118 (10.5%) could not be contacted, either because they had migrated or had died. Of the 987 subjects included in this study, 49.6% were men. A majority of them were illiterate (81.6%), living in joint families (82.9%), belonging to a low socioeconomic status family (48.8%), living with the spouse and children (56%). Women were more likely to be illiterate (99% vs. 63.9%), widowed (49.7% vs. 20.4%), living alone (4.2% vs. 1.0%), having son as the head of the household (51.3% vs. 27.8%) and unemployed (74.4% vs. 54.5%).

## RESULTS

### Tobacco smoking

Of the 987 respondents, 27.8% had never smoked, 56.5% were current smokers and 15.7% were past smokers (Table 1). The prevalence of smoking was high in males compared to females (71.8% vs. 41.4%) and this difference was statistically significant (p<0.001). Only 5.1% of the men and 5.4% of the women were current tobacco chewers, Less than 1% of the subjects consumed tobacco in other forms, i.e. tooth powder/paste or snuff.

**Table 1:** Prevalence of tobacco and alcohol use among study subjects

Pattern of use	Men (*n*=490) *n* (%)	Women (*n*=497) *n* (%)	Total (*n*=987) *n* (%)	p
**Tobacco smoking**				
Never smokers	60 (12.2)	214 (43.1)	274 (27.7)	<0.001
Ever-smokers			
Past smokers	78 (15.9)	77 (15.5)	155 (15.7)	0.85
Current smokers	352 (71.8)	206 (41.4)	558 (56.5)	<0.001
**Tobacco chewing**			
Never chewers	460 (93.9)	462 (93)	922 (93.4)	0.56
Ever-chewers			
Past chewers	5 (1)	8 (1.6)	13 (1.3)	0.42
Current chewers	25 (5.1)	27 (5.4)	52 (5.3)	0.82
**Alcohol**			
Never users	363 (74)	491 (98.8)	854 (86.5)	<0.001
Ever-users			
Past users	47 (9.6)	2 (0.4)	49 (5)	<0.001
Current users	80 (16.3)	4 (0.8)	84 (8.5)	<0.001

*Note*: (i) Some of the respondents used both tobacco and alcohol

(ii) Tobacco includes tobacco-containing products such as tooth powder/paste and snuff

### Alcohol use

The majority of the respondents had never consumed alcohol in their life (86.5%), 8.5% were current users and 5% were past users (Table 1). Around 16% of the men compared to <1% of the women were current alcohol users and this difference was found to be statistically significant (p<0.001). The remaining 37.2% (21.2% of the men and 52.9% of the women) were not using either tobacco or alcohol and the difference between men and women was statistically significant (p<0.001).

The majority of the current users of alcohol were occasional users (*n*=59; 74%) and they consumed 50–250 ml of alcohol on each occasion; only 21 of them (26%) were regular users. The prevalence rate among women was only 0.8%, so no further analysis was undertaken.

### Age and prevalence of tobacco and alcohol use

The proportion of subjects smoking tobacco declined from 77% in the 60–64 years age group to 63.9% in the ≥75 years age group in men (p<0.001), and from 45.8% in the 60–64 years age group to 32.9% in the ≥75 years age group in women (p<0.05) (Table 2). The proportion of men consuming alcohol also declined with increasing age from 25.4% in the 60–64 years age group to 10.5% in the ≥75 age groups. This trend was also found to be statistically significant (p<0.001). However, no such trend was observed in women.

**Table 2:** Gender- and age-specific prevalence of tobacco smoking and alcohol consumption

	Males			Females		
		
Age group (years)	*n*	Smoking *n* (%)	Alcohol *n* (%)	*n*	Smoking *n* (%)	Alcohol *n* (%)
60–64	126	97 (77)	32 (25.4)	179	82 (45.8)	1 (0.6)
65–69	114	88 (77.2)	21 (18.4)	133	58 (43.6)	0
70–74	117	82 (70)	13 (11.1)	103	39 (37.9)	2 (1.9)
≥75	133	85 (63.9)	14 (10.5)	82	27 (32.9)	1 (1.2)
Total	490	352 (71.8)	80 (16.3)	497	206 (41.4)	4 (0.8)

### Level of smoking in relation to age and sex

A majority of the men smokers were light to moderate smokers (41.5% and 42.9%,respectively) and only 15.6% were heavy smokers (Table 3). The proportion of heavy smokers decreased from 22.7% in the 60–64 years age group to 8.2% in the ≥75 years age group. However, in light smokers, there was an increase from 27.8% in the 60–64 years age group to 51.8% in the ≥75 years age group. This linear trend was also found to be statistically significant (p<0.001). The women were mainly limited to light smoking (71.8%) and no definite linear trend was observed (p=0.78).

**Table 3:** Level of current smoking according to age

	Men			
	
Age group (years)	*n*	Light *n* (%)	Moderate *n* (%)	Heavy *n* (%)
60–64	97	27 (27.8)	48 (49.5)	22 (22.7)
65–69	88	36 (40.9)	38 (43.2)	14 (15.9)
70–74	82	39 (47.6)	31 (37.8)	12 (14.6)
≥75	85	44 (51.8)	34 (40.0)	7 (8.2)
Total	352	146 (41.5)	151 (42.9)	55 (15.6)

		Women			
	
60–64	82	59 (72.0)	20 (24.4)	3 (3.7)
65–69	58	44 (75.9)	13 (22.4)	1 (1.7)
70–74	39	25 (64.1)	10 (25.6)	4 (10.3)
≥75	27	20 (74.0)	6 (22.2)	1 (3.7)
Total	206	148 (71.8)	49 (23.8)	9 (4.4)

*Note:* Light, moderate and heavy smokers defined as those who smoked ≤5, 6–20 and >20 cigarettes/*beedis* daily.

### Reasons for quitting tobacco and alcohol among past users

The most common reason for quitting tobacco smoking was doctor's advice, i.e. 37% for the entire group. Other important reasons included advice by relatives and friends (29%), some illness (cough: 15%, asthma: 9%, giddiness: 6%). Religious and financial reasons were quoted as a cause by only 6% and 4%, respectively.

The most common reason for quitting alcohol among men was self-desire and advice by relatives or friends (45%), followed by religious reasons (25%), doctor's advice (13%) and financial constraints (8%).

The relationships between current use of tobacco and alcohol with selected variables were examined (Table 4). A higher proportion of the literates, very healthy individuals and those with no history of any chronic morbidity were reported to be smoking and consuming alcohol. The differences in the parameters studied were found to be statistically significant except socioeconomic status. The type of family had a significant difference in relation to smoking but not alcohol (Table 4).

**Table 4:** Differential of smoking and alcohol use according to various sociodemographic variables

		Smoking		Alcohol	
			
Variable	*n*	Yes *n* (%)	No *n* (%)	Yes *n* (%)	No *n* (%)
Literacy					
Illiterate	805	438 (54.4)	367 (45.6)	51 (6.3)	754 (93.7)
Literate	182	120 (65.9) (p=0.005)	62 (34.1)	33 (18.1) (p<0.001)	149 (81.9)
Socioeconomic status					
Upper	158	87 (55.1)	71 (44.9)	14 (8.9)	144 (91.1)
Middle	347	199 (57.3)	148 (42.7)	25 (7.2)	322 (92.8)
Lower	482	272 (56.4) (p=0.89)	210 (43.6)	45 (9.3) (p=0.055)	437 (90.7)
Type of family					
Nuclear	169	108 (63.9)	61 (36.1)	20 (11.8)	149 (88.2)
Joint	818	450 (55.0) (p=0.034)	368 (45.0)	64 (7.8) (p=0.09)	754 (92.2)
Self-rated health					
Very healthy	126	85 (67.5)	41 (32.5)	18 (14.3)	108 (85.7)
Quite healthy	203	119 (58.6)	84 (41.4)	26 (12.8)	177 (87.2)
Not healthy	658	354 (53.8) (p=0.014)	304 (46.2)	40 (6.1) (p=0.001)	618 (93.9)
Chronic morbidity					
Present	821	447 (54.4)	374 (45.6)	63 (7.7)	758 (92.3)
Absent	166	111 (66.9) (p=0.003)	55 (33.1)	21 (12.7) (p=0.036)	145 (87.3)

*Note:* The height and weight could not be taken in 5 respondents.

The effect of addiction on the nutritional status of the respondent was studied (Table 5). Non-smokers and non-alcoholics had chronic energy deficiency (CED) compared to smokers (50.7% vs. 57%). The CED was 62.2% in tobacco and alcohol users and 47.0% in those who were only alcoholics, but these differences were not found to be statistically significant.

**Table 5:** Effect of addiction on the nutritional status of the study subjects (n=982)

Addiction	*n*	CED (<18.5) *n* (%)	Normal (18.5–25.0) *n* (%)	Overweight (>25.0) *n* (%)
Only smoking	489	279 (57.0)	195 (39.9)	15 (3.1)
Only drinking	17	8 (47.0)	7 (41.2)	2 (11.8)
Smoking and alcohol	66	41 (62.2)	23 (34.8)	2 (3.0)
Non-smokers and non-alcoholics	410	208 (50.7)	176 (42.9)	26 (6.3)

*Note:* The height and weight could not be taken in 5 respondents. CED: chronic energy deficiency

Among those consuming neither alcohol nor tobacco, 6.3% were overweight compared to 3.1% among those using tobacco but not alcohol and the difference was found to be statistically significant (p=0.02). When the group consuming neither of the two substances was compared with those currently consuming alcohol with or without smoking (3.0% and 11.8%, respectively), the differences were not statistically significant, probably due to the small numbers in these two groups (p=0.44 and p=0.7, respectively).

## DISCUSSION

A high prevalence of smoking (57%) was seen in the present study. A high prevalence of smoking is a common phenomenon among men in rural India^5^,^6^,^10^,^11^ and other developing countries.^12^–^14^

It was observed during the study that sharing *hookah* among the elderly, especially in evenings at the village *chaupal* (a common place in a village) is a socially accepted source of social interaction and recreational pursuit. Smoking is also used to extend hospitality to friends and visitors. Another aspect of smoking is that those who work in fields, smoke *beedi* when they rest in between and interact with each other, and the habit is carried to later life.

In this study, more men compared to women were smokers (72% vs. 41%). Other studies^6^,^11^ have also reported a higher prevalence of smoking in men than in women. Informal discussions in the community revealed that though men start smoking at an early age, women start the habit at a later age, (generally after childbirth). Thus, it appears that smoking in elderly women is socially acceptable.

The present data show that smoking was more in the age group of 60–64 years (77% and 45.8% in men and women, respectively) and reduced to 63.9% among men and 32.9% among women in the ≥75 years age group. The possible reasons for the declining prevalence of tobacco with age might be cessation of smoking by the elderly due to respiratory problems. This could also be due to the bias introduced by higher survival of non-smokers or higher mortality among smokers.

In the present study, 8.5% of the elderly were currently consuming alcohol. Other studies^6^,^10^ have also reported a similar prevalence of alcohol use in this population, while few studies from other countries reported a prevalence varying from 5% to 28%.^12^–^14^ A rural Iowa study^15^ reported a very high prevalence of alcohol use among the elderly (51%). These differences may be due to variations in cultural acceptance.

In the present study, 16.3% of the men and 0.8% of the women consumed alcohol. The National Family Health Survey (1998–99) in a combined rural–urban data found that 20% of elderly men and 0.5% of elderly women consumed alcohol in Haryana, whereas at the all India level, 18.6% of elderly men and 3.1% of elderly women consumed alcohol. The Iowa study also reported a higher prevalence of alcohol use among elderly men than elderly women (61% vs. 41%). Swaddiwudhipong *et al*.^12^ have reported that 20.3% of men and 5.3% of women consume alcohol. This gender difference could again be due to social reasons, e.g. men have peer groups and drinking alcohol was socially accepted. This practice was acceptable only among elderly women belonging to particular ethnic groups.

The prevalence of alcohol use declined from 25.4% in the 60–64 years age group to 10.5% in the ≥75 years age group among men. Young^14^ has also reported similar findings. The reason could be similar to those of tobacco use/smoking.

A higher proportion of the elderly who were smokers and alcohol users belonged to a nuclear family, did not have any chronic morbidity, and had a low body mass index (BMI). This study, being cross-sectional, cannot attribute any direction to this relationship. However, available evidence suggests that cigarette smoking leads to a lower BMI.^16^

## CONCLUSION

Tobacco use has been socially accepted among adults and the elderly in most Indian societies. The finding of a high prevalence of smoking/alcohol consumption in elderly rural men of India is of serious concern, and therefore needs some remedial measures. We should create an environment in the community that help smokers who want to quit, and those who quit should continue to persuade and help other smokers to quit. Awareness should be made of the ill effects of smoking and alcohol use by individual and group discussions at the community level through health workers and through the media (at the national level) and encourage adopting healthy lifestyles. Besides health professionals, religious *gurus* may be powerful agents for influencing change of smoking and drinking behaviours in rural areas. We too need to learn about the factors that have an influence on cessation. Medical assistance should be provided to those wishing to quit smoking to overcome the withdrawal effects. Apart from familial support, some cessation services should be provided to people who are not able to gather sufficient support from outside or within themselves to quit the habit and sustain it.
